# The effects of real and simulated microgravity on cellular mitochondrial function

**DOI:** 10.1038/s41526-021-00171-7

**Published:** 2021-11-08

**Authors:** Hong Phuong Nguyen, Phuong Hoa Tran, Kyu-Sung Kim, Su-Geun Yang

**Affiliations:** 1 Inha Institute of Aerospace Medicine, Inha University College of Medicine, Incheon, 22332 Korea; 2 Department of Biomedical Science, BK21 FOUR Program in Biomedical Science and Engineering, Inha University College of Medicine, Incheon, 22332 South Korea; 3 Department of Otorhinolaryngology, Head and Neck Surgery, Inha University Hospital, Incheon, 22332 South Korea

**Keywords:** Cell biology, Molecular medicine

## Abstract

Astronauts returning from space shuttle missions or the International Space Station have been diagnosed with various health problems such as bone demineralization, muscle atrophy, cardiovascular deconditioning, and vestibular and sensory imbalance including visual acuity, altered metabolic and nutritional status, and immune system dysregulation. These health issues are associated with oxidative stress caused by a microgravity environment. Mitochondria are a source of reactive oxygen species (ROS). However, the molecular mechanisms through which mitochondria produce ROS in a microgravity environment remain unclear. Therefore, this review aimed to explore the mechanism through which microgravity induces oxidative damage in mitochondria by evaluating the expression of genes and proteins, as well as relevant metabolic pathways. In general, microgravity-induced ROS reduce mitochondrial volume by mainly affecting the efficiency of the respiratory chain and metabolic pathways. The impaired respiratory chain is thought to generate ROS through premature electron leakage in the electron transport chain. The imbalance between ROS production and antioxidant defense in mitochondria is the main cause of mitochondrial stress and damage, which leads to mitochondrial dysfunction. Moreover, we discuss the effects of antioxidants against oxidative stress caused by the microgravity environment space microgravity in together with simulated microgravity (i.e., spaceflight or ground-based spaceflight analogs: parabolic flight, centrifugal force, drop towers, etc.). Further studies should be taken to explore the effects of microgravity on mitochondrial stress-related diseases, especially for the development of new therapeutic drugs that can help increase the health of astronauts on long space missions.

## Introduction

### Microgravity and spaceflight research

Since the first human spaceflight in 1961, the effects of space stress on the human body have been studied and reported in many ways. Astronauts are exposed to harsh extreme space environment other than microgravity, such as cosmic radiations, tight work schedules, broken circadian rhythms, isolation, and more. During the early stages of space exploration, concerns regarding the effects of spaceflight on the human body comprised a wide range of physical functions, including visual acuity, fluid redistribution, immune and nervous system regulation, bone and muscle loss, and kidney stone formation^1–5^. Further, these space stresses affect the health of astronauts in complex ways.

One of the most notable space stresses is “weightlessness,” which involves body fluid redistribution^2^. Astronauts encounter the body fluid shift, typically represented by cephalic fluid redistribution from the lower limbs to the rest of the body, at the beginning of spaceflight. In addition, scientists suspect that space microgravity is the main cause of cascadic changes of the physiological and biological functions in the human body^4,6^. Microgravity may also affect the health and normal cellular homeostasis of a person. Various forms of evidence exist to suggest that oxidative stress increases under different conditions that are experienced during microgravity^7,8^. However, due to financial and time constraints, there are not many opportunities to conduct in-depth research on organisms and on humans to understand their biological processes and underlying mechanisms during spaceflight. For that reason, it is complicated to interpret human experimental results, which should be read also in the light of individual diversity. It is even harder to compare them with data obtained in vitro. Therefore, a crucial challenge for scientists is to create microgravity conditions on Earth that can realistically simulate the microgravity environment of space. To date, numerous ground-based instruments and models have been developed, including two-dimensional clinostats, random positioning machines, rotating wall vessels, diamagnetic levitation, hind limb unweighting (HLU), tail-suspension models, and head-down bed rest models, thereby allowing more flexible research and experiments to be scheduled, controlled, modified, and repeated at low costs^9–11^.

### Mitochondria and its metabolism

Mitochondria, the membrane-bound organelles that are present in most eukaryotic cells, play a critical role in cellular function and dysfunction, including calcium signaling, cell growth and differentiation, cell cycle control, and cell death. The occurrence of mitochondrial dysfunction has been implicated in various metabolic and age-related disorders, neurodegenerative diseases, and ischemic injury within the heart and brain^12^. As a byproduct of electron transfer during adenosine triphosphate (ATP) production, mitochondria produce intracellular reactive oxygen species (ROS), such as superoxide anions (O_2_^−^), hydroxyl radicals (OH^−^), and hydrogen peroxide (H_2_O_2_), with extra electrons from the electron transport chain reacting with oxygen to produce O_2_^−^^13,14^. However, O_2_^−^ can be detoxified by superoxide dismutase (SOD), a mitochondria-specific antioxidant, resulting in the formation of H_2_O_2_. H_2_O_2_ can then be converted to water by glutathione (GSH) peroxidase (GPx); however, if unquenched, H_2_O_2_ can form the highly reactive OH^−^ by reacting with metal ions. In addition to cellular sources of ROS, environmental mutagens can also induce ROS production^13,15,16^. Although it remains unclear whether mitochondrial dysfunction or damage to the organelles results in increased ROS levels, it is well-known that increased ROS levels are associated with pathological disorders^17–22^. Therefore, over the past decade, mitochondria have become an attractive therapeutic target to deliver new treatments, including antioxidants.

Oxidative stress related to microgravity conditions has been studied in various tissues and cells, including the hippocampus^23^, the brain cortex^24^, neural stem cells^25^, the ocular tissue^26^, and the intestine^27^. This review outlines the mechanism through which real and simulated microgravity (SM) affects mitochondrial oxidative stress and explores this mechanism in different target tissues.

## Effect of microgravity on organs and cellular mitochondria

### Mitochondrial oxidative stress caused by microgravity in human organs and tissue

**Fig. 1:**
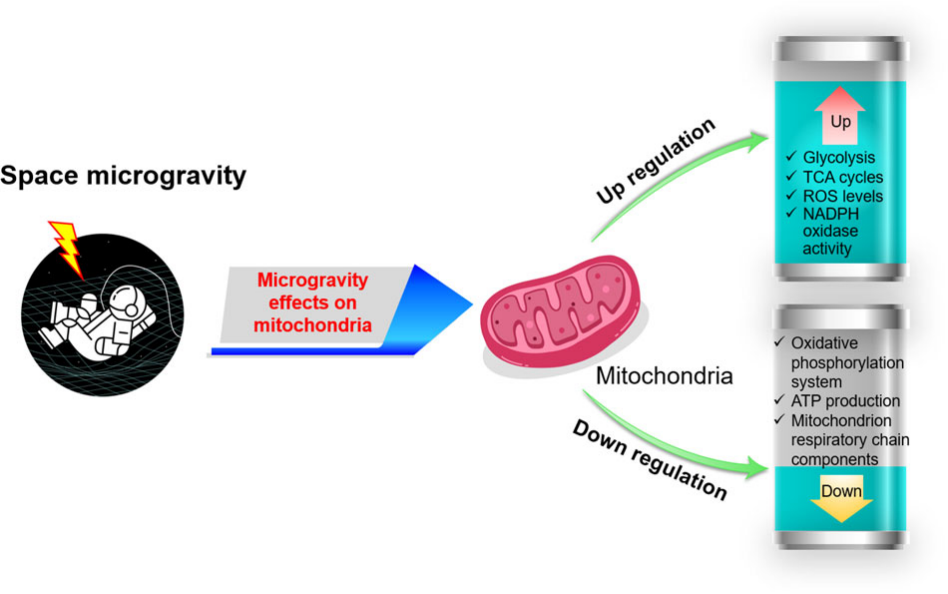
Space microgravity and biological functions of mitochondria. Space microgravity induces the upregulation of glycolysis, TCA cycles, ROS levels, and NADPH oxidase activity in mitochondria. On the other hand, oxidative phosphorylation, ATP production, mitochondrion respiratory chain components are downregulated. (Copyright of spaceman icon was purchased from The Noun Project, Inc. CA, https://thenounproject.com/term/spaceman/854150/).

Previously, research by Garrett-Bakelman et al.^28^, mentioned that at least ten key physiological processes were influenced by long-duration spaceflight, which serve as targets for development of countermeasure interventions during future exploration class human space travel. These include body mass and nutrition, ocular structure, vascular health, and cognitive function. Others also stated that microgravity induces a number of significant physiological changes in ocular tissue, the cardiovascular, nervous, immune systems, as well as the bone tissue of astronauts^29^. Here we will mainly focus on the effects of spaceflight or/and microgravity on key physiological parts targeting mitochondrial functions in human organs. To sum up, microgravity affects biological function of mitochondria resulting in the upregulation of glycolysis, tricarboxylic acid cycles, ROS levels, and nicotinamide adenine dinucleotide phosphate (NADPH) oxidase activity but in the downregulation of oxidative phosphorylation system, ATP production, and mitochondrial respiratory chain components (Fig. 1).

### Ocular system under spaceflight microgravity

Oxidative stress increases retinal damage and degeneration. Furthermore, recent studies support the finding that ROS, within the ocular tissue, are associated with various pathological conditions, including age-related degeneration, diabetic retinopathy, and cataracts^25,30–32^. Under microgravity conditions, cephalad fluid shift occurs, which causes an increase in intraocular pressure and possible changes in retinal vascular diameter^33^. Intraocular pressure increases by 58% during parabolic flights; however, considerable changes to the caliber of retinal arteries, demonstrating the stress exerted on the eye, have not been observed^33^. Yang et al.^34^ noted a correlation between spaceflight and oxidative stress related to lipids and DNA, and Mao et al.^26^ reported a significant increase in the apoptosis of photoreceptor cells of mice (taken aboard a space shuttle flight) compared to control mice that remained on Earth. They found that the spaceflight environment induces mitochondrial oxidative damage in the ocular tissue, which can result in retinal apoptosis in the inner nuclear and ganglion cell layers. In space, the expression of mitochondria-associated apoptotic genes was altered in vivo. In addition, genes responsible for ROS production were considerably upregulated in spaceflight samples. Furthermore, lipid peroxidation-specific 4-hydroxynonenal protein levels were significantly increased in the retina, implying a high risk of retinal degeneration to astronauts resulting from spaceflights.

### Neural system under HLU or SM condition

Microgravity has been known to cause cardiovascular deconditioning and various studies have suggested its correlation with cerebrovascular oxidative stress injury^35–42^. However, whether mitochondrial dysfunction is implicated in vascular oxidative stress injury during microgravity and whether differential mitochondrial dysfunction exists in HLU remains to be determined. Recently, Zhang et al.^43^ reported that mitochondrial dysfunction during microgravity may cause cerebrovascular oxidative injury. Using HLU to stimulate the effects of microgravity on rats, differential structural and functional changes were observed to occur in the cerebral and mesenteric arteries^35,44^, showing a significant increase in mitochondrial ROS levels, mitochondrial permeability transition pore opening, and malondialdehyde content, which is a sensitive marker of lipid peroxidation^43^. However, the abundance of mitochondrial MnSOD/GPx-1 decreased in HLU rat cerebral arteries but not in mesenteric arteries. Zhang et al.^45^ demonstrated that mitochondria under SM regulate the expression and activity of NADPH oxidases. Therefore, SM can cause cerebrovascular mitochondrial dysfunction by regulating the crosstalk between NADPH oxidase and mitochondria.

Wang et al.^23^ used a rat tail-suspension model to study the effects of SM on metabolic proteins in the hippocampus, and 42 and 67 mitochondrial metabolic proteins were found to be differentially expressed after 7 and 21 days of exposure to SM, respectively. Furthermore, mitochondrial complexes I, III, and IV were downregulated, in addition to the expression of isocitrate dehydrogenase and malate dehydrogenase (MDH). However, the expression of DJ-1 and peroxiredoxin 6, which protect cells against oxidative damage, was upregulated in the hippocampus. The mass spectrometry results were confirmed by western blot analysis of DJ-1 and cytochrome c oxidase 5A proteins. Despite these changes in mitochondrial protein expression after 21 days of exposure to SM, no apparent cellular apoptosis was observed. Therefore, the results of this study indicate that SM-induced oxidative stress affects metabolic protein expression.

Proteins, fats, and sugars (the primary energy sources of mammalian cells) are utilized through well-known biochemical mechanisms, which have been observed and studied under normal gravity conditions (terrestrial gravity). Espinosa-Jeffrey et al.^46^ reported that SM affects both energy and lipid metabolism in oligodendrocytes (OLs), the myelin-forming cells that insulate neuronal axons in the central nervous system. Increased mitochondrial respiration and increased glycolysis in human embryonic brain-derived neural stem cells were observed 24 h after exposure to SM. Moreover, by examining the secretome of OLs for 3 days after exposure to SM, they confirmed an increase in the Krebs cycle flux. This study also revealed that microgravity significantly increased the synthesis of fatty acids and complex lipids, such as 1,2-dipalmitoyl-GPC (5.67), and lysolipids, such as 1-oleoyl-GPE (4.48). Although long-chain lipids were not observed in this study, it is possible that with time, OLs would have progressed toward synthesizing lipids that constitute myelin.

### Hematologic and immune system

#### Hematologic and immune system under spaceflight microgravity

Spaceflight inhibited T-cell activation in returning Apollo astronauts, which was thought to be caused by hormonal changes^47,48^. It was also documented that mitogenic stimulation in weightlessness decreased lymphocyte proliferation^23,25^. Furthermore, human lymphocytes under spaceflight microgravity and/or SM showed mitochondrial clustering and morphological alterations in mitochondrial cristae when observed under a transmission electron microscope after 4 and 48 h of exposure^49^. Jurkat cells subjected to space shuttle flight have displayed abnormalities in microtubule organizing centers, increase in glucose metabolism, and time-dependent and microgravity-related increase in apoptosis antigen 1 (FAS/APO-1) protein expression, which is involved in apoptosis^50^.

#### Hematologic and immune system under SM condition

Under microgravity conditions, increased apoptosis of human lymphocytes has been observed^49^. Similar results were observed in *Drosophila* Schneider S-1 cells during clinostat rotation. Under SM, mitochondria were evenly distributed in the cytoplasm at 48 h, even though the abnormal morphology of the mitochondrial cristae persisted. Accumulation of mitochondria under SM is more pronounced toward one side of the cell, which indicates microtubule network disruption. Concurrent observation has revealed microtubule disruption and the failure of mitochondrial transport along the microtubules in human lymphocytes, resulting in increased apoptosis as exhibited via DNA fragmentation.

#### Cardiac and skeletal muscle system

Under microgravity conditions, movement patterns and muscular forces are markedly altered, and the functional, morphological, and biochemical properties of both human and rodent skeletal muscles undergo changes^51^. Unfortunately, limited information is available regarding the mechanism through which the heart responds to microgravity.

#### Cardiac and skeletal muscle system under spaceflight microgravity

The effects of gravity on the cardiovascular system during spaceflight have not yet been fully addressed; however, the blood volume that normally pools in the lower limbs under normal gravity conditions accumulates in the thorax, thereby temporarily increasing venous blood volume in return^39–42^. This shift in blood volume may be induced by utilizing pathways similar to those involved in stretch-induced cardiac hypertrophy that causes alterations in gene expression^52^. Connor et al.^53^ examined the expression of nuclear and mitochondrial genes in cardiac and skeletal muscles (triceps brachii) in response to short-duration exposure to microgravity. The study demonstrated a significant 32% increase in the enzyme activity of heart MDH along with a 62% elevation in heart MDH mRNA levels. Despite slight increase in the levels of mRNAs encoding subunits III, IV, and VIc, and a 2.2-fold increase in subunit IV protein content after exposure to microgravity, the enzymatic activity of COX was unaffected. In skeletal muscle, although MDH expression was unaffected after exposure to microgravity, COX activity reduced significantly by 41%, whereas subunit III, IV, and VIc mRNA levels and subunit IV protein levels remained unchanged. Thus, in response to microgravity, tissue-specific (i.e., heart vs. skeletal muscle) differences in the regulation of nuclear-encoded mitochondrial proteins were observed. In addition, the expression of nuclear-encoded proteins, such as COX subunit IV and MDH, was differentially regulated within a tissue.

#### Cardiac and skeletal muscle system under SM condition

Recently, Locatelli et al.^54^, by using a combination of mass spectrometry-based approaches, compared the relative abundance and turnover rates of 848 and 196 proteins, respectively, in rat neonatal cardiomyocytes exposed to SM and normal gravity. According to gene functional enrichment analysis, the protein content and function of the mitochondria, ribosomes, and endoplasmic reticulum were differentially modulated under microgravity. The experiments confirmed that protein synthesis decreased, whereas apoptosis, cell viability, and protein degradation remained largely unaffected under microgravity. These data support their conclusion that under microgravity conditions, cardiomyocytes attempt to maintain mitochondrial homeostasis at the expense of protein synthesis.

#### Cardiovascular system under SM condition

Endothelial cells, known as the gatekeepers of vascular integrity and function, are crucial for providing continuous perfusion such that the tissues can sustain their own processes. Several studies have demonstrated that SM can affect the function of human umbilical vein endothelial cells^8,55^. In human primary endothelial cells, Locatelli et al.^54^ showed that microgravity induces mitophagy, which contributes to endothelial adaptation for gravitational unloading. After 4 and 10 days of exposure to SM in a rotating wall vessel, the amount of BCL2-interacting protein 3, a marker of mitophagy, increased, and mitochondrial content, oxygen consumption, and maximal respiratory capacity were all simultaneously reduced, suggesting the acquisition of an efficient phenotype to meet the novel metabolic challenges created by gravitational unloading. Moreover, they suggested that microgravity-induced disorganization of the actin cytoskeleton triggered mitophagy, thereby creating a connection between cytoskeletal dynamics and mitochondrial content with gravitational unloading.

#### Skeletal system under spaceflight microgravity and/or SM condition

In vivo, the most severe effects of microgravity on astronauts are related to the loss of bone mass and osteopenia that accompany extended spaceflight^6,56^. The response of human primary osteoblasts exposed to SM was studied and the results of metabolomic and proteomic profile analyses revealed a prominent dysregulation of mitochondrial homeostasis^57^. Mitochondrial protein levels decreased upon gravitational unloading treatment, thereby primarily affecting the efficiency of respiratory chain. Metabolomic analysis results revealed that several metabolic pathways were influenced by microgravity through the stimulation of glycolysis and the pentose phosphate pathways, and disruption of the Krebs cycle during succinate–fumarate transformation. Furthermore, the results of proteomic analysis revealed that Complex II of the mitochondrial respiratory chain was downregulated by 50%. Accordingly, the expression of quinones 9 and 10 was downregulated. Complex III was upregulated by 60%, whereas Complex IV was downregulated by 14% with a reduction in the proton transport-coupled ATP synthesis. Finally, an oxidative stress response, induced by microgravity treatment, indicated a significant decrease in oxidized GSH and antioxidant enzymes. Decrease in MDH levels reversed the malate–aspartate shuttle, which contributed to the dysregulation of ATP synthesis. β-Oxidation of fatty acids was also inhibited, thereby promoting triglyceride production, while reducing the glycerol shuttle. Therefore, these findings suggest that microgravity may suppress bone cell functions by impairing mitochondrial energy potential and the overall energy state of the cell.

#### Hair tissues under spaceflight microgravity

Hair is known as the usefulness of hair follicles in the study of biogenesis^58^. The effects of long-term exposure to the microgravity environment of space on astronauts were investigated by analyzing hair samples of ten astronauts who were on the International Space Station (ISS) for 6 months^59^. Two samples each from before (preflight), during (inflight), and after (postflight) the missions on the ISS were collected. Quantitative PCR was used to analyze the ratios of mitochondrial to nuclear DNA (nDNA) and RNA. The combined preflight, inflight, and postflight data revealed a significant reduction in the mitochondrial to nDNA ratio for inflight and significant reductions in the mitochondrial to nuclear RNA ratio for both the inflight and postflight samples. Except for the postflight samples, the mitochondrial RNA to DNA ratios were relatively constant. The expression of the following redox and signal transduction-related genes was also examined using the same sample: MnSOD, CuZnSOD, Nrf2, Keap1, GPx4, and catalase. The results of the combined preflight, inflight, and postflight data showed a significant decrease in the expression of all redox-related genes for the postflight collected samples, except for catalase, which showed no change. Therefore, this decreased expression may contribute to increased oxidative stress inflight, resulting in postflight mitochondrial damage.

#### Cancer cells under SM condition

Microgravity is known to influence major cellular processes such as the cell cycle, proliferation, and differentiation^42–46^. Several studies have reported the anticancer potential of microgravity through cell growth inhibition^47–49^. SM inhibits cellular mitochondrial activity due to the deceleration of mitosis in human malignant glioma^50^. Recently, the anticancer mechanism of microgravity that induces autophagy via mitochondrial dysfunction in human Hodgkin’s lymphoma (HL) cells was reported^51^. After 2 days of exposure to SM, human HL cells increased their ROS production and NADPH oxidase family gene expression, whereas a decrease in their mitochondrial mass and ATPase, ATP synthase, and intracellular ATP levels was observed. Furthermore, human HL cells exposed to SM underwent autophagy via AMPK/Akt/mammalian target of rapamycin (mTOR) and mitogen-activated protein kinase (MAPK) pathway modulation; however, the ROS scavenger, *N*-acetylcysteine (NAC), inhibited this autophagy. These results suggest a new therapeutic approach to HL, which is different from conventional chemotherapeutic and radiotherapeutic approaches.

Increased estrogen receptor (ER) expression regulates mitochondrial oxidative stress under SM in human breast cancer cells^52^. Intracellular oxidative stress and its underlying mechanisms were investigated using a rotary cell culture system to achieve an SM environment. Experiments were conducted using human breast cancer cell lines—MCF-7 (an ERα-positive cell line) and MDA-MB-231 (an ERα-negative cell line)—encapsulated in alginate/collagen carriers. Exposure to SM for 48 h resulted in oxidative stress and DNA damage in MDA-MB-231 cells; however, a significant increase in mitochondrial activity along with minimal DNA damage in MCF-7 cells was observed. SOD activity significantly increased in MCF-7 cells and decreased in MDA-MB-231 cells under SM conditions compared with a standard gravity control. Moreover, SM promoted ERα and protein kinase C (PKC) epsilon expression in MCF-7 cells treated with the PKC inhibitor, Gö6983. Overall, exposure to SM increased mitochondrial activity in ERα-positive cells, but caused cellular oxidative damage in ERα-negative cells. Thus, under SM conditions, ERα may play an important role in protecting cells from oxidative stress damage.

#### Proposed mechanism of ROS accumulation in mitochondria under microgravity

**Fig. 2:**
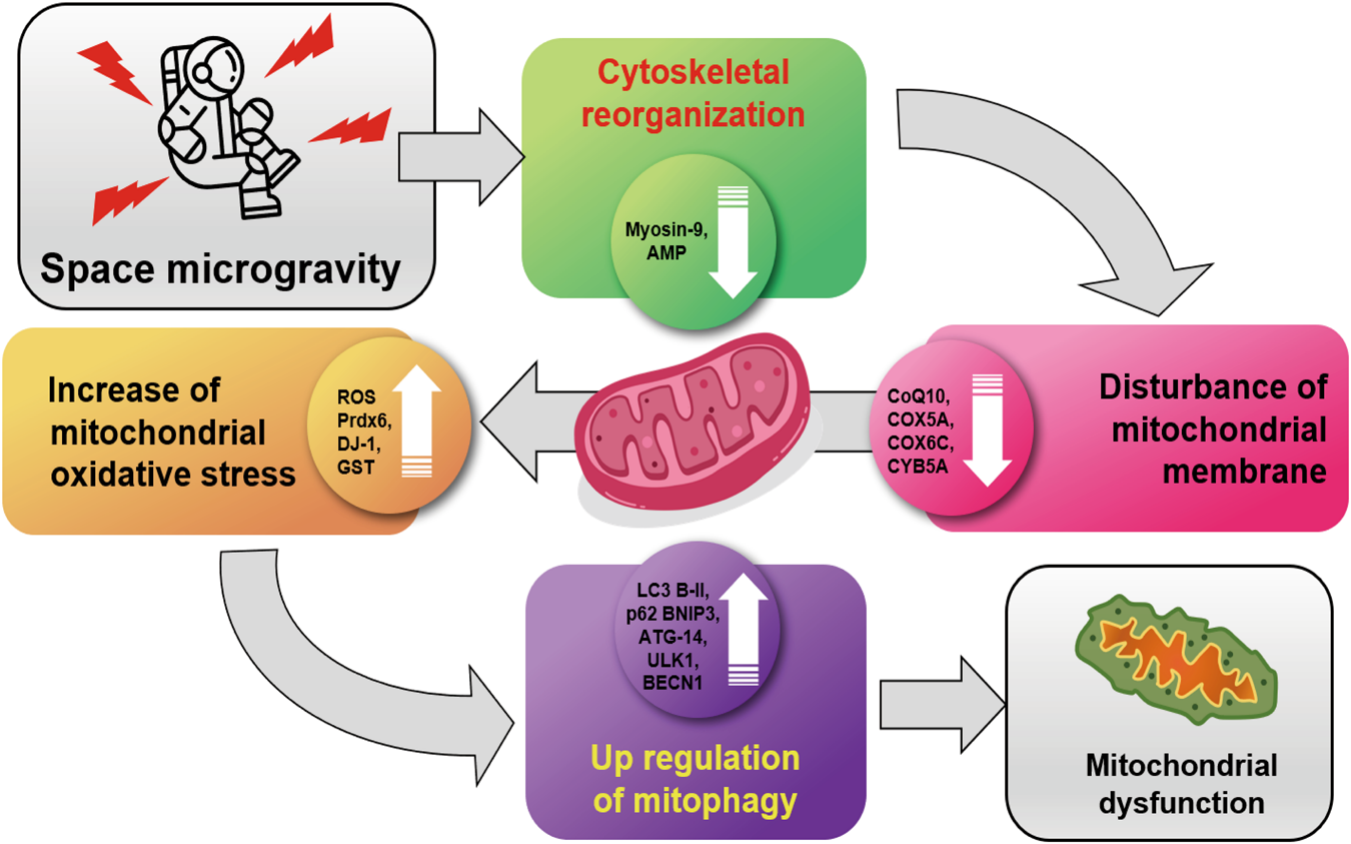
Space microgravity and mitochondrial dysfunction. Space microgravity causes cytoskeletal reorganization with the decrease of muscle markers such as Myosin-9 and AMP. Cytoskeletal disruption affects mitochondrial membrane with the decrease of antioxidant factors (CoQ10, COX5A, COX6C, and CYB5A) and the increase of mitochondrial oxidative stress that triggers mitophagy and reduces mitochondrial content and dysfunction. (Copyright of spaceman icon was purchased from The Noun Project, Inc., CA, https://thenounproject.com/term/spaceman/854150/).

The effects of microgravity on mitochondrial stress have been widely reported. However, the mechanism underlying ROS induction under microgravity remains a mystery. Given the difficulty of carrying out experiments in space with multiple stress factors, experimental protocols on the ground-based models or SM have been developed to study the mechanism of the effects of weightlessness. Cells depend on microtubules for their structure and the transport of organelles for cell division. Adhesion sites on the surface of the cell also incorporate microtubules and actin into their structure^60^. Therefore, changes in cell structure will affect the environmental response of the cells. Observable changes in mitochondrial clustering and the area around the nuclear envelope are likely caused by alterations to the cytoskeleton^61^. Mitochondrial clustering may increase cellular glucose consumption. Previous studies have reported how cellular mitochondrial distribution and the cytoskeletal network are of critical importance to mitochondrial function and movement^62,63^. The cytoskeletal disruption in SM leads to increased mitophagy, thereby typically resulting in the decrease of mitochondrial mass (Fig. 2)^54^. In vitro assay revaled exposure of SM increased ROS production and NADPH oxidase gene expression but decreased ATPase, ATP synthase, and intracellular ATP levels^64^. An increase in cell apoptosis is a notable consequence of microgravity that affects cell structure and function^26,49^. Also, in vivo studies performed during spaceflight has been shown to distort the expression of cytoskeletal proteins, which disturb the distribution of mitochondria in rat gastrocnemius muscles^65^.

**Fig. 3:**
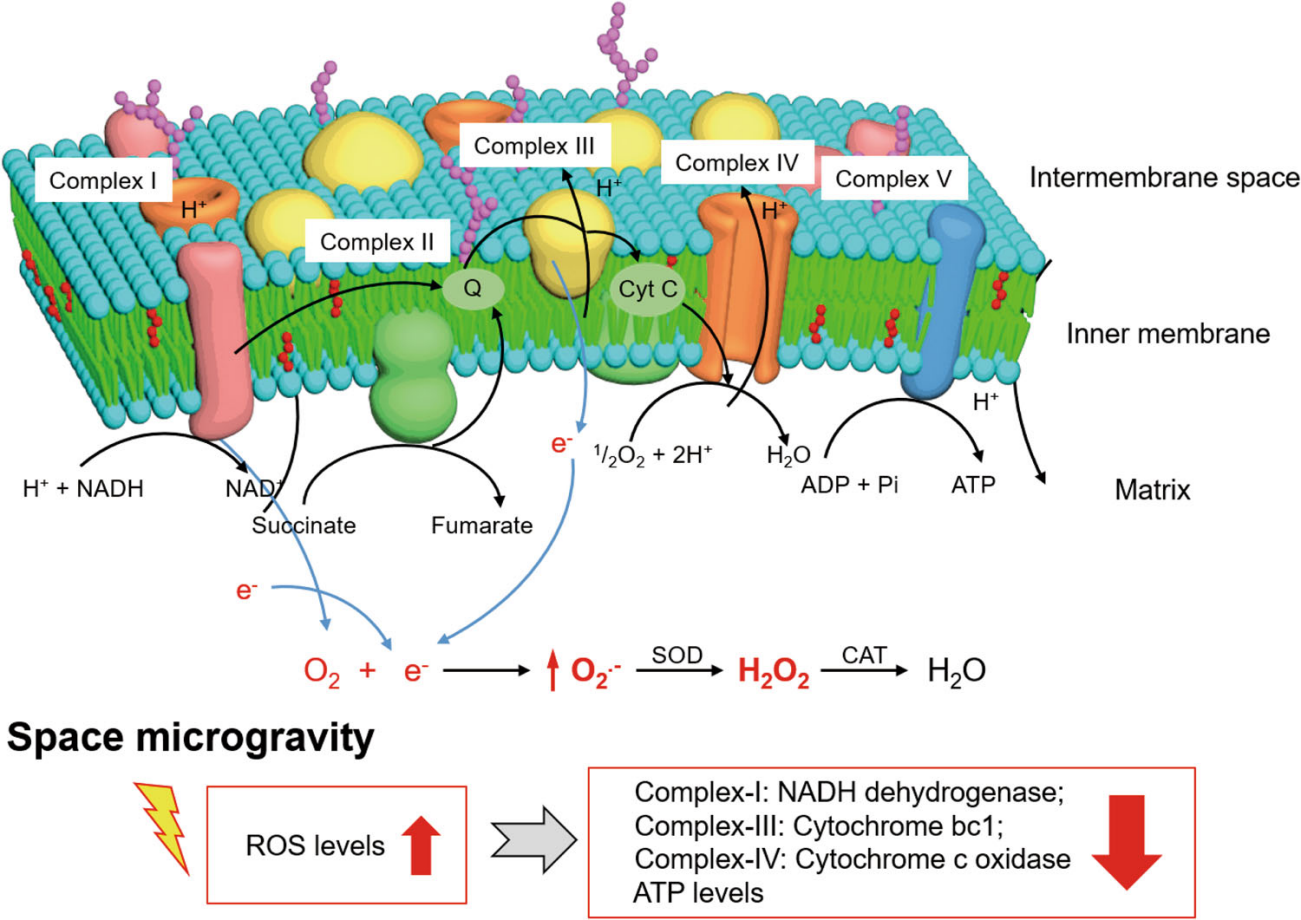
ROS generation and electronic transport chain of mitochondria in microgravity environment. Complex I, Complex III, and Complex IV are downregulated. Mitochondrial ROS, including O_2_^−^, OH^−^, and H_2_O_2_ increase under microgravity condition. O_2_^−^ are mainly produced by Complex I and III, and reduced to H_2_O_2_. Molecular oxygen may take electrons from Complex III to form ROS. The reduced activity of cytochrome *c* oxidase leads to the downregulated levels of ATP.

An imbalance between ROS production and antioxidant defense causes oxidative stress. The accumulation of ROS can damage proteins, lipids, and DNA^66^. ROS can cause genomic damage, resulting in mutations that can lead to cell transformation^67^. Mitochondria are the primary sources of ROS production, which affect important cellular functions, including oxidative stress, oxidative phosphorylation, and apoptosis^68^. Maintaining proper mitochondrial function is a critical aspect of cellular defense against oxidative stress. Proteomic analysis has demonstrated mitochondrial membrane dysfunction that results from the impairment of other mitochondrial respiratory chain components under microgravity conditions^57^. A small fraction of electrons leaves the electron transport chain during reactions between Complex III and IV, and the premature electron leakage generates O_2_^−^ in the presence of oxygen (Fig. 3). Instead of producing membrane-impermeable O_2_^−^, Complex III produces the membrane-permeable superoxide, HOO^69^, which can easily reach the cytosol as it can be released into both the mitochondrial matrix and the intermembrane space^70^.

#### Molecular strategies for targeting antioxidants in mitochondria

Mitochondrial health is a key moderator of cellular function including the regulation of cellular survival, orchestration of anabolic and metabolic pathways, as well as ROS signaling. Exercise is one of the most potent therapeutic approaches for the improvement of mitochondrial content, quality oxidative phosphorylation, and respiratory capacity per mitochondrion^71–73^. Endurance exercise training can increase the total mitochondrial volume per volume of muscle fiber by up to 43% in female and by 37% in male subjects^74^. Moreover, Place et al.^75^ reported that the production of ROS was attenuated after chronic training. Exercise is widely used as a countermeasure to microgravity environment^76,77^. Dorfman et al.^78^ found that women who exercised only 45 min per day can prevent the cardiac atrophy after 60 days of bed rest. Exercise training may be a useful intervention to alleviate skeletal muscle atrophy after hind limb suspension in mice^79^.

**Fig. 4:**
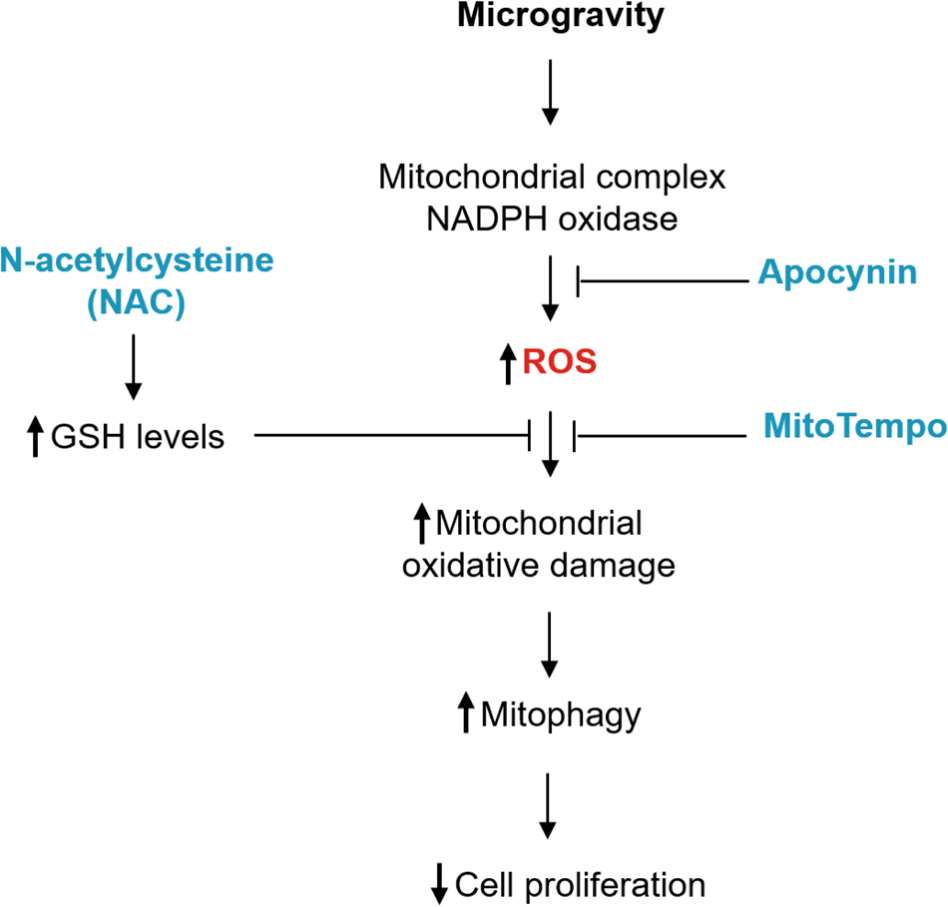
Molecular strategies for targeting antioxidants to the mitochondria. MitoTempo increases mitochondrial O2^−^ dismutation and accumulates in the mitochondria. Apocynin, an NADPH oxidase inhibitor, attenuates ROS production as well as the resultant neuroinflammation and mitochondrial injury. *N*-acetylcysteine (NAC) is an aminothiol and synthetic precursor of intracellular cysteine and GSH, and is thought to be an important antioxidant.

A number of natural supplements have been used to treat mitochondrial dysfunction, including vitamins, minerals, antioxidants, metabolites, enzyme inhibitors and cofactors, mitochondrial transporters, and herbs^80–82^. Antioxidants to restore mitochondrial function, in terms of mitochondrial ROS scavengers, has been studied for a long time and has shown reliable results. Basically, researchers may confer that these antioxidants could be applied to the field of space research with the equivalent effects on mitochondria, even though these antioxidants have not been studied on the real space microgravity condition. We have listed some antioxidants with favorable mode of action for the mitochondrial molecular activity (Table 1 and Fig. 4). However, if any antioxidant erratically exhibits superior or inferior activity in space, scientists still have not enough data to explain any specific mechanism or molecular pathways for this variation.

**Table 1:** Reported antioxidants applicable for space mitochondria stress.

Name	Synonyms	Mechanism	Ref
MitoQuinone	- Mitoquinone cation - Plastoquinonyl-decyl-triphenylphosphonium - MitoQ	- Mitochondria-specific target design (triphenylphosphonium) of Coenzyme Q10 - Increasing the electron transporter activity between NADH dehydrogenase and succinate dehydrogenase	^90^
Methylene blue	- Methylthioninium chloride - 3,7-Bis(dimethylamino)phenothiazin-5-ium chloride	- Increasing the activity of complex IV ROS scavenging	^91–93^
Resveratrol	- 3,5,4′-Trihydroxy-*trans*-stilbene - 3,4′,5-Trihydroxystilbene	- Upregulating mitochondria-located antioxidant enzymes ROS scavenging	^94^
SkQ1	- Plastoquinonyl-decyl-triphenylphosphonium - Visomitin	- Mitochondria-specific target design (triphenylphosphonium) of plastoquinone - Increasing the ratio of anti- vs. pro-oxidant concentrations	^95^
Lipoic acid	- 6,8-Dithiooctanoic acid - Tioctic Acid - Thioctanoic acid - Berlition	- A cofactor for mitochondrial 2-ketoacid dehydrogenases	^96^
*N*-acetylcysteine	- Acetylcysteine - Mercapturic acid	- Increasing the intracellular GSH levels	^97^

MitoTempo, a mitochondria-targeted SOD antioxidant mimetic, increases mitochondrial O_2_^−^ dismutation and accumulates in the mitochondria; however, MitoTempo does not affect cytoplasmic dismutation in endothelial cells^83^. Apocynin, an NADPH oxidase inhibitor, attenuates ROS production and results in neuroinflammation and mitochondrial injury^84^. As these reports demonstrate crosstalk between NADPH oxidase and mitochondria in a microgravity environment, treatments to promote the recovery of mitochondrial function in HLU rat cerebral arteries has been suggested using the NADPH oxidase inhibitor, apocynin, and mitochondria-targeted antioxidant, mitoTempol^43,45^.

NAC is an aminothiol and synthetic precursor of intracellular cysteine and GSH, and is thought to be an important antioxidant. NAC can increase intracellular GSH levels, owing to its antioxidative or free radical scavenging property; however, NAC also has a reducing property due to its thiol-disulfide exchange mechanism^85^. Recently, the anticancer mechanism of microgravity that induces autophagy via mitochondrial dysfunction in human HL cells was reported^64^. After 2 days of exposure to SM, human HL cells increased their ROS production and NADPH oxidase family gene expression, whereas a decrease in their mitochondrial mass and ATPase, ATP synthase, and intracellular ATP levels was observed. Furthermore, human HL cells exposed to SM underwent autophagy via AMPK/Akt/mTOR and MAPK pathway modulation; however, NAC inhibited this autophagy. These results suggest a new therapeutic approach to HL, which is different from conventional chemotherapeutic and radiotherapeutic approaches.

In summary, antioxidants directly targeting mitochondrial O_2_^−^ scavenging prevent an increase in mitochondrial ROS production and reveal potential novel therapeutic development that may greatly improve the health of astronauts.

## Conclusion

In general, microgravity/SM affects mitochondrial dysfunction in all cell types by increasing mitochondrial ROS levels, by causing an imbalance of mitochondrial gene expression, and by damaging DNA (Tables 2 and 3). Spaceflight-induced microgravity clearly showed that the DNA mitochondria damage was caused by significant reductions in the mitochondrial DNA (mtDNA)/nDNA ratios, by alterations to mitochondria-associated apoptotic genes, and by ROS-mediated gene upregulation^26,59^. Ground-based facilities or models that produce SM, therefore, might mimic the specific effects of microgravity on humans or other organisms. The results showed that SM also induces mitochondrial imbalance by increasing mitochondrial respiration, by glycolysis and Krebs cycle flux, and by decreasing of mitochondrial mass and ATP levels^7,43,46,54^.

**Table 2:** Reported mitochondrial stress under space or simulated microgravity.

Species	Type of subject	Type of study	Facility	Exposure time	Changes	Ref
Female C57BL/6 mice	Ocular tissue	In vivo	Space	13 Days	Mitochondria-associated apoptotic genes altered ROS-mediated genes upregulated Apoptotic HNE protein upregulated	^26^
Human and *Drosophila*	1. Human lymphocytes (Jurkat) 2. *Drosophila melanogaster (*Schneider S-l) cells	In vitro	Space and Clinostat	20 h	Displayed mitochondria abnormalities increased	^49^
Rat	Cerebral arteries	In vivo	Hind limb unweighting (HLU)	4 Weeks	Mitochondrial ROS levels, mPTP opening, and MDA content increased Mitochondrial MnSOD/GPx-1 levels decreased Cerebrovascular mitochondrial dysfunction and crosstalk between NADPH oxidase and mitochondria	^43^
Human	Fetal cortical tissue	In vitro	SM	1–5 Days	Increased mitochondrial respiration and increased glycolysis 24 h after exposure to SM Increased the Krebs cycle flux Increase in the synthesis of fatty acids and complex lipids, such as 1,2-dipalmitoyl-GPC (5.67), and lysolipids, such as 1-oleoyl-GPE (4.48)	^46^
Human	Human umbilical vein endothelial cell	In vitro	SM	4–10 Days	BCL2-interacting protein 3 expression, a marker of mitophagy, increased Mitochondrial content, oxygen consumption, and maximal respiratory capacity reduced → microgravity-induced disorganization of the actin cytoskeleton triggered mitophagy	^54^
Rat	Cerebral and mesenteric arteries	In vivo	HLU	4 Weeks	Superoxide levels increased in cerebral arteries mRNA levels of *Nox2* and *Nox4* upregulated NADPH oxidases activated	^45^
Rat	Cardiac and skeletal muscles	In vivo	SM	6 Days	Increase in heart malate dehydrogenase (MDH) Heart cytochrome *c* oxidase (COX) enzyme activity remained unchanged Skeletal muscle COX activity significantly reduced	^53^
Rat	Neonatal rat cardiomyocyte	In vitro	SM	12–120 h	Microgravity protein synthesis decreased; however, apoptosis, cell viability, and protein degradation largely unaffected.	^98^
Human	Hodgkin’s lymphoma cell	In vitro	SM	2 Days	Increased ROS production and NADPH oxidase family gene expression Mitochondrial mass and ATPase, ATP synthase, and intracellular ATP levels decreased	^64^
Rat	Hippocampus	In vivo	Rat tail-suspension model	7–21 Days	Mitochondrial complexes I, III, and IV downregulated, in addition to isocitrate dehydrogenase and MDH expression DJ-1 and peroxiredoxin 6 expression upregulated No obvious cell apoptosis observed	^23^
Human	Breast cancer cell lines (MCF-7 and MDA-MB-231)	In vitro	SM	48 h	Oxidative stress and DNA damage Superoxide dismutase (SOD) activity significantly increased in MCF-7 cells but decreased in MDA-MB-231 cells Increased mitochondrial activity in ERα-positive cells but induced cellular oxidative damage in ERα-negative cells	^99^
Human	Malignant glioma cell	In vitro	SM	3 Days	Mitochondrial activity inhibited in the control group	^100^
Human	Primary osteoblasts	In vitro	SM	5 Days	Decrease in the expression of mitochondrial proteins, respiratory chain stimulating glycolysis, and the pentose phosphate pathways Krebs cycle interrupted Complex II of the mitochondrial respiratory chain, downregulated by 50%. Complex III upregulated by 60%, whereas Complex IV downregulated by 14%	^57^
Human	Hair samples	In vivo	Space	6 Months	Significant reduction in the mtDNA/nDNA ratios Reduction in the mtRNA/nRNA ratios Decrease in the expression of all redox-related genes	^59^
Roundworm	*Caenorhabditis elegans*	In vitro	SM	24 h	Activation of mtUPR by the ATP-binding cassette protein, HAF-1, and the homeodomain-containing transcriptional factor, DVE-1 Increase in HSP-6 and HSP-60 expression	^101^

**Table 3:** Reported the altered mitochondrial markers under space or simulated microgravity.

Molecular function enrichment	Species/target	Molecular class/term	Facility	Upregulation	Downregulation	Ref
A mitochondrial Mn-superoxide dismutase (Mn-SOD)	Nematode *Caenorhabditis elegans*	Gene expression	SM		*sod-3*	^102^
Mitochondria-associated gene expression	Mice/ocular cell	Gene expression	Spaceflight	*Bak1*, *Bcl2l1*, *Bid, Fxc1*, *Opa1*, *Pmaip1*, *Sh3gl*, *Slc25a13*, *Slc25a16*, *Slc25a17*, *Trp53*, *Tspo*, *Ucp1*, *Ucp2*	*Slc25a37*	^26^
Intracellular mitochondrial genes	Human neural stem cells	Protein expression	SM	NRF1, NRF2, and Tfam,		^103^
Proteins in the mitochondrial respiratory chain	Mice/skeletal muscles	Protein expression	Spaceflight	Ndufs4, ATP6v1b2, Cox6c	Atp5b, Etfa, Coq6, Cox5a, Cox5b, Etfdh, Ndufa10, Nudfa9, Ndufa10, Nudfb9, Ndufb10, Ndufs1, Ndufs2, Ndufs3, Ndufv1, Ndufv2, Samm50	^104^
Other mitochondrial proteins			Spaceflight		Mfn1, Mtfp1	^104^
NADPH oxidase activity	Rat/arteries	Protein expression	SM	Nox2, Nox4		^45^
Kreb’s cycle	Human/osteoblasts	Protein expression	SM		ODO2, SUCB1, FUMH, SDHA, MDHM	^57^
Glycolysis			SM	LDHA, PGAM1	TPI1, G3P, ENOA	^57^
PPP			SM	6PGD	TKT	^57^
Ubiquinolcytochrome-*c* reductase activity			SM	UQCRC1, NQO1, CYB5R3	UCRI	^57^
Cytochrome *c* oxidase activity			SM		COX5A, CYB5A, COX6C	^57^
Antioxidant activity			SM	PRDX4, PRDX5, PRDX6, SODC	PRDX1, PRDX2, PRDX3	^57^
Oxidative stress-related genes	Human hair	Gene expression	Spaceflight		*MnSOD*, *CuZnSOD*, *Nrf2*, *Keap1*, *GPx4*	^59^
Glycolysis	Human embryonic brain	Metabolomic Profile	SM	Lactate levels	Glucose, pyruvate levels	^46^
TCA cycle			SM	Citrate levels, Aconitate, fumarate, malate levels		^46^
Enzyme activity	Rat cardiac and skeletal muscles	Gene expression	Spaceflight	*Mdh*, *Cox*		^53^
Oxidative phosphorylation (OXPHOS), glycolysis, and TCA proteins.	Rat hippocampus	Metabolomic profile	SM	Dihydrolipoamide *S*-acetyltransferase, glucose phosphate isomerase, MDH1, NAD (soluble), oxoglutarate (α-ketoglutarate) dehydrogenase (lipoamide), phosphofructokinase, muscle, phosphoglycerate mutase 1 (brain), and pyruvate kinase isozyme M2	NADH dehydrogenase (ubiquinone) flavoprotein 2 (Ndufv2); ATPase, H+ transporting, lysosomal V1, subunit B2 (Atp6v1b2); ATPase, H+ transporting, lysosomal V1, subunit E1 (Atp6v1e1); and ATP synthase, H+ transporting, mitochondrial Fo complex, subunit F6 (Atp 5j); glutamate dehydrogenase; and Complex I: NADH dehydrogenase, Complex III: Cytochrome bc1, and Complex IV: COX	^23^
Oxidative stress markers			SM		MDA and H_2_O_2_	^23^
NADPH oxidase family genes	Human Hodgkin’s lymphoma cells	Gene expression	SM	NADPH oxidase family genes (gp91-, p22-, p47-, and p67-phox)	ATPase (ATP1A1) and ATP synthase (ATP5A1)	^64^
Autophagy-related genes			SM	ULK1, ATG14, BECN1, and LC3		^64^
Phosphorylation			SM	ULK1, ATF4, Beclin-1, and microtubule-associated protein 1 light chain 3 (LC3)		^64^
Inhibit autophagy			SM	LKB1 and activated AMPK	Bcl-2 family proteins Bcl-2 and Mcl-1	^64^
Mitochondrial proteins	Human umbilical vein endothelial cell (HUVEC)	Protein expression	SM		MTCO, VDAC, and CYP D	^54^
Markers of autophagy			SM	LC3 B-II and p62		^54^
Marker of mitophagy			SM	BNIP3		^54^
Lipid oxidation	Rat cerebral arteries	Protein expression	SM	MDA		^43^
Antioxidant			SM		MnSOD and GPx	^43^
Mitochondrial protein/gene	*Caenorhabditis elegans*	Protein expression	Spaceflight		SDHA-1, GPD-3, PCK-1, GEI-7, ACO-2, CTS-1	^105^
TCA cycle		Gene expression	Spaceflight		*pdha-1*, *C04C3.3*, *dld-1*, *cts-1*, *aco-1*, *aco-2*, *idha-1*, *idhb-1*, *idhg-1*, *idhg-2*, *idh-2*, *gei-7*, *dlst-1*, *dld-1*, *sdha-1*, *sdhb-1*, *mev-1*, *sdhd-1*, *fum-1*, malate dehydrogenase 1 (*mdh-1*)	^105^

In this review, we mainly focus on effects of the microgravity and SM on cellular mitochondrial functions. However, as there are other stressors associated with spaceflight, radiation exposure is another primary factor of stress in space that could significantly impact mitochondrial functions by increasing ROS levels^27,86,87^. The high energy of space radiation may contribute significantly to increased health risks for astronauts^88^. DNA damage is the main effect of radiation exposure^89^. Hair samples from ten astronauts who stayed aboard the ISS for long durations revealed that the mtDNA/nDNA ratio was decreased when comparing preflight and inflight samples, and the mtRNA.nRNA ratio also decreased for inflight and postflight when compared to preflight^59^. Although a reduced number of cells recovered in postflight, this implies that the DNA in the hair follicles were damaged due to space radiation, but the damage, in terms of mtDNA transcription, remained comparable to that of postflight.

Results from microgravity or SM experiments have established that mitochondrial dysfunction is one of the consequences of exposure to space. A few publications have shown that mitochondria activate an adaptive response mechanism to cope with a microgravity environment^28,54^. Human umbilical vein endothelial cell (HUVEC) exposure to SM, which was generated by rotating the wall vessel, increases autophagy, which leads to mitophagy and loss of mitochondrial mass. The decreased mitochondrial mass protects cells from an overproduction of ROS and HUVEC cells acquire a thrifty phenotype to reach a new equilibrium that maintains cell survival and fundamental functions^54^. In “the NASA twin study,” mitochondria dysfunction was also observed through the mitochondrial-related changes at genomic and functional levels after 1 year in space^28^. Higher levels of mtRNA during the inflight were detected and compared with preflight and postflight. The extracellular flux assay showed a decrease in mitochondrial respiration and in spare reserve ATP capacity. The authors proposed that these changes under conditions of microgravity is an adaptive mechanism of mitochondria experiencing the harsh conditions of the new environment. They also observed that some of these alterations are reversible upon return to normal gravity, including mitochondrial mass, mtRNA levels, and lactic acid levels^28,54^.

This review demonstrates that weightlessness, whether actual or simulated, has significant effects on mitochondrial functions, which can lead to notable adaptive changes or to an apoptotic response in cells. In general, studies have suggested that mitochondria undergo a reduction in size when exposed to microgravity. After a period of time, restructuring of the cytoskeleton disrupts the mitochondrial structure, thereby reducing mitochondrial content, oxygen consumption, and respiratory capacity. However, scientists in this field still lack sufficient data or information to determine whether real microgravity, especially in deep space, causes mitochondrial stress in the same way as observed on Earth. Scientists are now more focused on space medicines that can prevent and/or relieve space stress. Thus, this review will contribute to a better understanding of the mechanisms involved in mitochondrial dysfunctions during prolonged exposure to microgravity, which will help researchers design appropriate precautions and contingencies to cope with space-related health issues.
